# Repeatable and heritable behavioural variation in a wild cooperative breeder

**DOI:** 10.1093/beheco/arx013

**Published:** 2017-02-18

**Authors:** Hannah A. Edwards, Terry Burke, Hannah L. Dugdale

**Affiliations:** a Department of Animal and Plant Sciences, University of Sheffield, Sheffield, S10 2TN, UK; b Nature Seychelles, PO Box 1310, Roche Caiman, Mahé, Republic of Seychelles; c Behavioural Ecology and Physiological Group, Groningen Institute for Evolutionary Life Sciences, University of Groningen, PO Box 11103, 9700 CC, Groningen, The Netherlands; d School of Biology, The Faculty of Biological Sciences, University of Leeds, Leeds, LS2 9JT, UK

**Keywords:** Cooperative breeding, exploration, personality, heritability, repeatability, Seychelles warbler

## Abstract

Quantifying consistent differences in behaviour among individuals is vital to understanding the ecological and evolutionary significance of animal personality. To quantify personality, the phenotypic variation of a behavioural trait is partitioned to assess how it varies among individuals, which is also known as repeatability. If pedigree data are available, the phenotypic variation can then be further partitioned to estimate the additive genetic variance and heritability. Assessing the repeatability and heritability of personality traits therefore allows for a better understanding of what natural selection can act upon, enabling evolution. In a natural population of facultative cooperatively breeding Seychelles warbler (*Acrocephalus sechellensis*) on Cousin Island, a lack of breeding vacancies forces individuals into different life-history strategies, and these differences in reproductive state could generate behavioural differences among individuals in the population. We used this population to estimate the repeatability of 4 behavioural traits (novel environment exploration, novel object exploration, obstinacy/struggle rate, and escape response), and narrow-sense heritability (of behavior, *h*^2^_B_; behavior minus observer variance; and personality), and evolvability, of the repeatable behavioural traits. We also tested for an among-individual correlation between the repeatable traits. We found that, compared to estimates in other study species, the exploratory behaviours were moderately repeatable (0.23–0.37), there was a positive among-individual correlation (0.51) between novel environment and novel object exploration, and that novel environment exploration was moderately heritable (0.17; *h*^2^_B_ was low as it includes observer variance). This study further clarifies the additive genetic variance available for selection to act upon in this cooperatively breeding bird.

## Lay Summary

To evolve and respond to natural selection behavioural traits must have a genetic component. Quantifying the genetic component of behaviour can thus reveal its evolutionary significance. In a cooperative breeder, we tested if behavioural traits were consistent, heritable and correlated. We found that our measures of exploratory behaviour were consistent and correlated, and that novel environment exploration, was heritable. This behaviour therefore has the greatest capacity to evolve in response to a changing environment.

## INTRODUCTION

Animal personality is a phenomenon where individuals exhibit consistent behavioural differences between one another (Biro and Stamps 2008; Smith and Blumstein 2008). Behavioural differences can occur in single or multiple traits, that can be categorised into axes such as shyness/boldness, exploration, aggression, sociability, and activity (Réale et al. 2007). When these axes are correlated they are known as behavioural syndromes, such as bold, aggressive and fast-exploring proactive strategies or shy, docile and slow-exploring reactive strategies (Coppens et al. 2010; Koolhaas et al. 1999; Sih et al. 2004a). From an adaptive perspective, the evolution of animal personality remains a puzzle because a plastic behavioural response would allow individuals to adapt to changing environments (Wolf et al. 2007). However, theoretical models suggest that personality could be generated and maintained if the fitness pay-offs associated with the behaviour were frequency-dependent (Wolf and McNamara 2012) or dependent on an individual’s properties or circumstances, known as state-dependence (Wolf et al. 2007; Sih et al. 2015).

To test the state-dependent model, studies of personality are required in systems where there are multiple states or life-history strategies. Breeding systems where 3 or more individuals help to raise offspring, such that at least one individual helps to raise offspring that are not their own, are known as cooperative breeding systems (Cockburn 1998). Individuals often help in their natal group rather than dispersing to gain their own breeding position (Wiley and Rabenold 1984). Cooperatively-breeding individuals can therefore adopt different states or life-history strategies, such as stay and help, stay and co-breed, stay and not help, or disperse and breed elsewhere. These differences in both reproductive and social state among individuals, which might be expected to favour a range of personalities, make cooperatively-breeding systems informative for investigating the evolution of personality.

Particular theoretical models that can be tested in cooperative breeding system include the asset protection and social state models (Wolf et al. 2007). When behaviour is dependent on asset protection, individuals with a high future reproductive state (i.e., individuals that invest in future reproduction or receive reproductive benefits in the future) are predicted to be consistently slow explorers and averse to risk, in order to reduce their risk of mortality, from predation compared with those that have a low future reproductive state (Dall et al. 2004; Stamps 2007; Wolf et al. 2007). For example, in the cooperatively-breeding Seychelles warbler, there is a correlation between personality and asset protection (Edwards et al. 2016). Furthermore, there is also the potential for personality to be dependent on social state. The social niche hypothesis suggests that socially living individuals that repeatedly interact with one another will benefit by developing social niches. Social niches, such as social status, cause individuals to behave differently by reducing social conflict and reinforcing consistency through positive feedback mechanisms such as learning (Bergmüller and Taborsky 2010; Wolf and Weissing 2010). The relationship between social state and personality is unclear; some studies have shown that a dominant social status correlates with fast exploration and aggressive and bold behaviour in a territorial context (Verbeek et al. 1996; Dingemanse and de Goede 2004; Favati et al. 2014), while other studies have found no such correlation (Gómez-Laplaza 2002; Fox et al. 2009; Edwards et al. 2016).

To understand how personality has evolved and how it has been maintained we must quantify its repeatability, heritability and evolvability. To quantify the repeatability of personality, individuals must be measured repeatedly for certain behavioural traits. From these repeat measures, the proportion of the total phenotypic variance (*V*_P_), that is explained by the difference between individuals (*V*_I_) can be estimated. Repeatability (*R*) can then be calculated as: *R = V*_I_*/ V*_P_ (Lessells and Boag 1987). Repeatability gives an indication of the consistency in the differences between individuals across contexts or over time (Bell et al. 2009). Over a range of taxa, on average 0.37 (SE = 0.01, *N* studies = 759, *N* taxa = 98) of the variance in behaviour has been shown to be accounted for by consistent differences among individuals (Bell et al. 2009).

Repeatability can be further partitioned to determine the genetic basis of personality and thus its potential evolutionary significance (Dingemanse 2002; Drent et al. 2003; Sinn et al. 2006; Kvarnström 2013, unpublished data). Narrow-sense heritability of behaviour (*h*^2^_B_) describes the proportion of the total phenotypic variance (*V*_P_) that can be explained by additive genetic variance (*V*_A_); *h*^2^_B_*= V*_A_*/ V*_P_ (Falconer and Mackay 1996). Across a range of species, 0.26 (SE = 0.01, *N* = 209) of the variance in behaviour has been shown to be accounted for by additive genetic variation (van Oers and Sinn 2013). However, a stricter way to estimate the proportion of personality variation attributable to additive genetic variation has been described by Dochtermann et al. (2015), whereby temporary environmental effects (e.g. measurement error) are excluded from *V*_P_. Using this heritability measure (*h*^2^_P_), on average, 0.52 (SE = 0.09, *N* = 70) of the variance in personality is explained by additive genetic variation (Dochtermann et al. 2015). This is important, as observer errors will differ between studies, thus hindering comparisons of heritability. Finally, evolvability (*I*_A_), the mean standardised additive genetic variance (*V*_A_ / trait mean^2^), is a further measure that allows comparison when using the same transformation across the same traits and populations with different means (Houle 1992).

An increasing number of wild population studies have quantified the additive genetic variance of personality in natural populations (Duckworth and Kruuk 2009; Blumstein et al. 2010; Taylor et al. 2012; Korsten et al. 2013; Poissant et al. 2013; Class et al. 2014; Petelle et al. 2015). By investigating the evolution of behaviour in natural populations, this prevents the artificial, controlled environment of the laboratory altering the expression of behaviour and selection on behavioural genetic variation (Weigensberg and Roff 1996; Archard and Braithwaite 2010). Consequently, heritability and repeatability estimates are often higher when sampled from natural rather than laboratory populations (Bell et al. 2009; van Oers and Sinn 2013). It is unclear whether captive/laboratory based personality assays do (Armitage et al. 1986; Boon et al. 2008; Fisher et al. 2015; Herborn et al. 2010; Svendsen and Armitage 1973) or do not (Boyer et al. 2010; Fisher et al. 2015) reflect behaviour in the wild.

To our knowledge, no study has investigated the heritability of personality in a natural population of a cooperatively breeding species, where different life-history strategies may play an important role in the evolution of personality. The Seychelles warbler (*Acrocephalus sechellensis*) provides an excellent opportunity to investigate personality in a cooperative breeding system. First, habitat saturation limits the number of available breeding territories, forcing some individuals to remain on their natal territory, instead of gaining a primary breeding position elsewhere, and help rear offspring that are not their own (Komdeur 1992). These differences in reproductive and social state among individuals could generate behavioural differences in the population (Bergmüller and Taborsky 2010; Wolf et al. 2012). Indeed, personality has been linked with asset protection in this system (Edwards et al. 2016). Second, there is little immigration and emigration between islands and the whole island population has been intensively monitored (Komdeur et al. 2004), so enabling the recapture of individuals for personality testing. Third, the population has a multi-generational genetic pedigree allowing the heritability of personality to be estimated. In this study, we investigated 4 potential personality traits in the population: exploration of a novel environment (e.g. Verbeek et al. 1994), exploration of a novel object (e.g. Verbeek et al. 1994), obstinacy/struggle rate (e.g. Réale et al. 2000) and escape response (e.g. Pérez et al. 2010). We quantified the repeatability of these 4 personality traits and then investigated the heritability and evolvability of the repeatable traits. We also quantified whether there were among-individual phenotypic correlations due to the association of similar traits in proactive versus reactive strategies (e.g. Koolhaas et al. 1999).

## METHODS

### Ethics statement

Local ethical regulations and agreements were followed for fieldwork. Nature Seychelles permitted us to work on Cousin Island Nature Reserve. The Seychelles Department of Environment and the Seychelles Bureau of Standards authorized fieldwork and sampling.

### Study system

The Seychelles warblers is an endemic facultative cooperative breeding species that now occurs on 5 islands within the Seychelles (Wright et al. 2014). Seychelles warblers on Cousin have been monitored closely since 1985 (Hammers et al. 2015). During summer (June–September) and most winter (January–February) breeding seasons individuals are monitored to identify territory boundaries (Richardson et al. 2001). Individuals are caught with mist nets, a metal British Trust for Ornithology (BTO) ring and colour ring are fitted if necessary, and a blood sample taken for molecular sexing (following Griffith et al. 2002) and parentage analyses. There is little migration of birds between islands, and, consequently, with the intense monitoring, there is a 0.98 ± 0.01 annual probability of re-sighting adults, enabling the accurate measurement of survival and reproduction (Brouwer et al. 2010).

The study island of Cousin (0.29 km^2^; 04°20′S, 55°40′E) has a carrying capacity of around 320 individuals that reside in ca. 115 territories (Komdeur and Pels 2005). Group membership was assigned to each bird (>5 months old) that was seen repeatedly on a territory interacting with group members and not exhibiting primary breeding pair behaviours (Richardson et al. 2002). A primary breeder status was assigned to individuals in a pair that were repeatedly seen in the same territory, stayed within close proximity, had constant vocal interactions with their mate and either mate guarded (if male) or were the object of mate guarding (if female). A territory generally contains a single primary breeding pair, and approximately 50% of territories also contain additional non-primary group-members (Kingma et al. 2016). Habitat saturation forces some individuals to assume non-primary roles, as helpers or non-helpers, because of the limited breeding vacancies (Komdeur 1992).

### Personality assays

We assayed 4 behaviours: obstinacy, exploration of a novel environment, exploration of a novel object and escape response. Supplementary Table S1 shows the sample sizes according to sampling intensity for each personality trait. Novel environment exploration was assayed throughout the summer of 2010 and the winter and summer breeding seasons of 2012–2015. Assays for the other 3 traits were conducted over shorter periods: obstinacy and escape response in 2010–2014, and exploration of a novel object in 2013–2015.

Individuals were caught by mist net; this is an active trapping strategy, focused on specific territories, that aims to capture target individuals. We believe this targeted capture strategy reduced any trapping bias that might be caused by individual behavioural differences, such that these differences would have limited impact on our sampling (Michelangeli et al. 2015). After being caught in a mist net, the individual was extracted, placed in a bird bag and suspended from a branch out of the wind. Obstinacy or struggle rate was then measured by counting the number of seconds of movement during 1 min in the bird bag (adapted from Réale et al. 2000).

After morphometric measurements were taken, individuals were rested for 5 min in a bird bag and then assayed for novel environment exploration following the methods in Edwards et al. (2015). Briefly, novel environment exploration was assayed in an Oxygen 4 tent (L322 × W340 × H210 cm; Gelert Ltd Wigan) containing 3 artificial trees (adapted from Verbeek et al. 1994). By observing through a small opening (15.24 cm wide by 6.35 cm tall) in the gauze of the tent door, the numbers of hops, flights and unique trees visited in 5 min were counted using tally counters, and totalled to give a measure of exploration (Edwards et al. 2015). Over the course of the sampling period, tent colour (blue/green), the orientation of the branches of the artificial trees (diagonal and parallel) and the way the bird was released into the tent (by hand or on to a tree) varied. These methodological factors were all controlled for in all statistical analyses.

After the novel environment assay, individuals remained in the tent and were given a 2-min break before the novel object assay (see acclimation test, Edwards et al. 2015). A novel pink toy attached to a tree branch (95 cm long) was inserted and positioned in the centre of the tent (adapted from Verbeek et al. 1994). For each bird, we initially included a control assay with the novel object excluded. The order of the novel object and control assays was randomised using a coin toss, with a 2-min gap between both trials. This control assay allowed us to test whether birds responded to the novel object or the stick, after which the control assay was no longer run (Edwards et al. 2015). The behaviour score (summed number of hops, flights, and trees visited in 5 min) was higher (Edwards et al. 2015), latency time (seconds to move once the assay had begun) was shorter (Wilcoxon signed rank test; *n* = 185, *V* = 3162, *P* < 0.001), and the number of stick touches was lower (Wilcoxon signed rank test; *n* = 185, *V* = 3162, *P* < 0.001) in the novel object assay than in the control assay. This confirmed that the behavioural reaction resulted from the novel object and not the stick to which it was attached (Supplementary Figures S1–3). Latency had very low repeatability (0.02, 95% credible Interval [Cr.I.] = 0.01–0.36, *n* = 177). Behaviour scores in the novel object assay were therefore used as a measure of exploration (Edwards et al. 2015).

Escape response was recorded back at the territory of capture. The departure time to fly from a man-made perch (consisting of a branch 24 cm in length attached to an 80-cm trunk) was recorded (adapted from Pérez et al. 2010). In pilot studies (*n* = 193 birds) the bird was placed in the palm of the hand, but this was changed to prevent hand temperature and movement affecting the measure. Therefore, the method change was accounted for in the analyses.

### Statistical analyses

All statistical analyses were performed in R 3.0.1. (R Development Core Team 2013) using MCMCglmm 2.17 (Hadfield 2009). For all univariate models, we specified an expanded prior: *V* = 1, *n* = 0.002, alpha.mu = 0 and alpha.V = 1000, because the variance was close to zero (Hadfield 2015). For the novel environment exploration univariate model, we specified an uninformative inverse gamma prior: *V* = 1 and *n* = 0.002. For the bivariate model we specified: *V* = diag (2), such that both variance priors were set at 1, and *n* = 1.002. Furthermore, for the observer identity random effect in the novel object exploration models, we specified the equivalent of a proper Cauchy prior: *V* = 1, *n* = 1, alpha.mu = 0, alpha.*V* = 25^2^ (Gelman 2006), due to having few observer levels (*n* = 7). We assessed convergence by inspecting the autocorrelation values (*r* < 0.1) and time-series plots of the model parameters, and using the heidel.diag and geweke.diag functions. Prior sensitivity analyses showed that our choice of priors had little influence on the results of the model (Supplementary Table S2). Power analyses (Morrissey and Wilson 2009) including informative individuals for the novel environment and novel object trait showed that we had enough statistical power (≥0.8) to detect heritabilities ≥0.24 and ≥0.26, respectively (Supplementary Figures S4 and 5).

### Repeatability

Generalised linear mixed models (GLMMs) were run using a Poisson distribution with log link for all traits except stress response, where a Gaussian distribution with identity link was used. The dependent variable was the personality trait. We tested fixed effects that we believe may be important and associated with personality in our cooperative breeding study system and have been associated with personality in other systems: social status (primary breeder or non-primary breeder, e.g. Bergmüller and Taborsky 2010), sex (e.g. Schuett and Dall 2009), time interval to next assay (days, e.g. Dingemanse et al. 2012), assay number (e.g. Dingemanse et al. 2012), season (number of days from 1 January to account for the minor breeding season; from 1 June to account for the major breeding season, e.g. Dingemanse 2002), year (only for obstinacy, stress response and escape response because year was collinear with tent colour/branch orientation/release method in the novel environment and novel environment exploration assays), body mass to account for body size (standardised for time of day, Quinn et al. 2011) and age (days, e.g. Fisher et al. 2015). Age and body mass were mean centred and divided by 2 standard deviations (Gelman and Hill 2006), and age was included as both a linear and quadratic term to model the non-linear relationship of senescence (Patrick and Weimerskirch 2015). Weather (sun, cloudy, partly cloudy, rain, sunset) and differences in the method used (tent colour, branch orientation and how the bird was released into the tent for novel environment exploration; tent colour and branch orientation for novel object exploration; release method for escape response) were also included. The random effects of bird identity and observer identity (obstinacy *n* = 13, stress response *n* = 7, novel environment exploration *n* = 11, novel object exploration *n* = 7, escape response *n* = 13) were included to account for multiple measures on the same bird and measures taken by different observers. The variance components were extracted from the GLMM, and the raw phenotypic repeatability of the personality trait captured following Nakagawa and Schielzeth’s (2010) calculation. The posterior distribution was sampled every 100 iterations, with a burn-in period of 3000 iterations and a run of 203000 iterations.

### Heritability

Parentage patterns are complex in the Seychelles warbler with ca 40% of offspring being fathered by a male from outside the natal territory (extra-group parentage, Hadfield et al. 2006). Furthermore, 2% of offspring were sired by non-primary within-group males, and 15% of offspring had mothers that were non-primary within-group co-breeders (Richardson et al. 2001; Hadfield et al. 2006). Parentage was assigned based on 30 microsatellites using Masterbayes 2.52 (Hadfield et al. 2006). The pedigree is 10 generations deep and contains 1853 individuals, of which 1809 were offspring. 786 individuals in the pedigree were informative for novel environment exploration and 684 were informative for novel object exploration (1487 offspring were assigned a mother and 1554 were assigned a father with at least 80% confidence; Dugdale et al. unpublished data).

We extended the univariate models from the repeatability analyses into “animal” models for the moderately repeatable traits following Kruuk and Hadfield (2007). The fixed effects were the same as in the repeatability analyses. The following random effects were added: an animal term, linked to the pedigree, to account for the additive genetic variance (*V*_A_); mother identity to account for the maternal effects (*V*_M_); individual identity to account for the permanent environment effects (*V*_PE_); and, observer identity to account for multiple measures by the same observer (*V*_O_). The variance components were extracted from the GLMM, and, extending Nakagawa and Schielzeth’s (2010) repeatability calculations for Poisson distributions, we then calculated: the heritability of behaviour (*h*^2^_B_) calculated as *h*^2^_B_*= V*_A_*/ V*_P_, the heritability of behaviour excluding observer variance (*h*^2^_B-O_), calculated as *h*^2^_B-O_ = *V*_A_ / (*V*_A_ + *V*_PE_ + *V*_M_ + *V*_Res_); and the heritability of personality (*h*^2^_P_) calculated as, *h*^2^_P_*= V*_A_/ (*V*_A_ + *V*_PE_ + *V*_M_), so as to exclude temporary environmental effects (Wilson et al. 2010; Dochtermann et al. 2015). We also calculated evolvabilities on the transformed scale as, *I*_A_ = *V*_A_*/trait mean*^2^, to get an indication of the expected change in the trait mean if subject to directional selection (Houle 1992). The posterior distribution was sampled every 500 iterations, with a burn-in period of 30,000 iterations and a run of 1,000,000 iterations.

### Behavioural correlation

To estimate among-individual correlation coefficients between the moderately repeatable traits, we ran a bivariate model with a similar structure to the univariate repeatability models. Correlations were calculated by dividing the covariance between the traits at the focal level by the square root of the product of the variance of the 2 traits. For the phenotypic bivariate model, the posterior distribution was sampled every 100 iterations, with a burn-in period of 3000 iterations and a run of 203,000 iterations.

## RESULTS

### Repeatability

**Figure 1:**
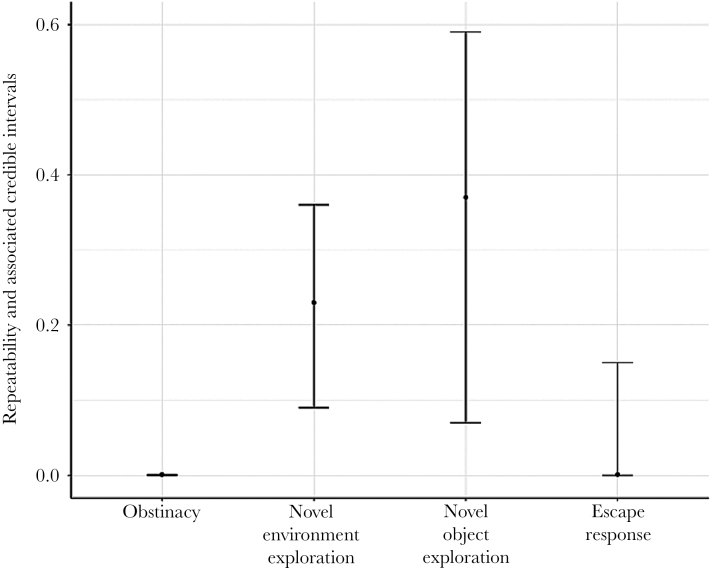
Repeatability estimates (posterior mode) for the 4 behavioural traits; error bars represent the 95% credible intervals.

Novel environment exploration and novel object exploration had moderate repeatability estimates (0.23 and 0.37, respectively, Figure 1). However, obstinacy and escape response had repeatability estimates close to zero (Figure 1).

**Figure 2:**
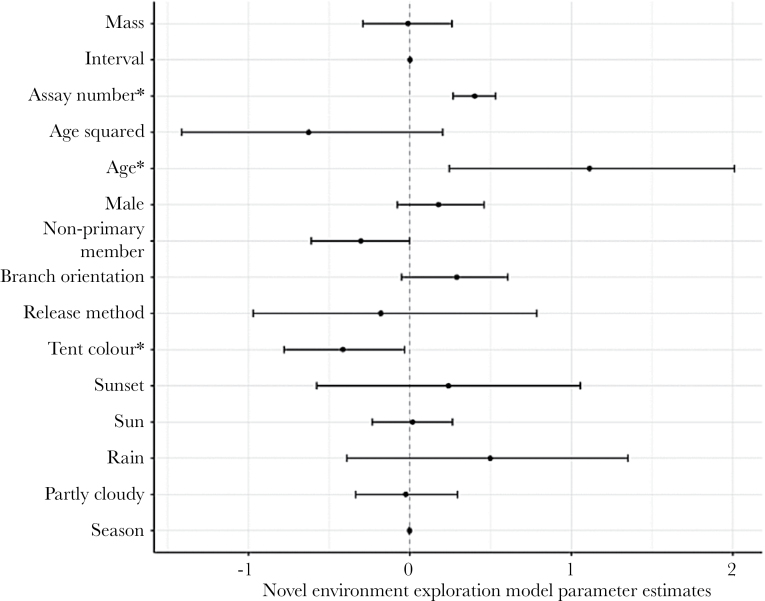
Posterior modes of the fixed effects, and associated 95% credible intervals, in the novel environment exploration model: mass (mean centred and divided by 2 standard deviations), interval (days between assay), assay number, age (mean centred and divided by 2 standard deviations; quadratic and linear terms), sex (male = 163, female = 149; contrast = female), social status (primary = 280, non-primary member = 237; contrast = primary), branch orientation (diagonal = 316, vs. parallel = 201; contrast = diagonal), release method (hand = 64, vs. placed on tree = 449; contrast = hand), tent colour (blue = 339, vs. gree*n* = 178; contrast = blue), weather (cloudy = 515, partly cloudy = 128, rain = 7, sun = 258, sunset = 9; contrast = cloudy), and season (number of days from the first of January or June).* indicates posterior modes where the 95% credible intervals do not overlap zero.

**Figure 3:**
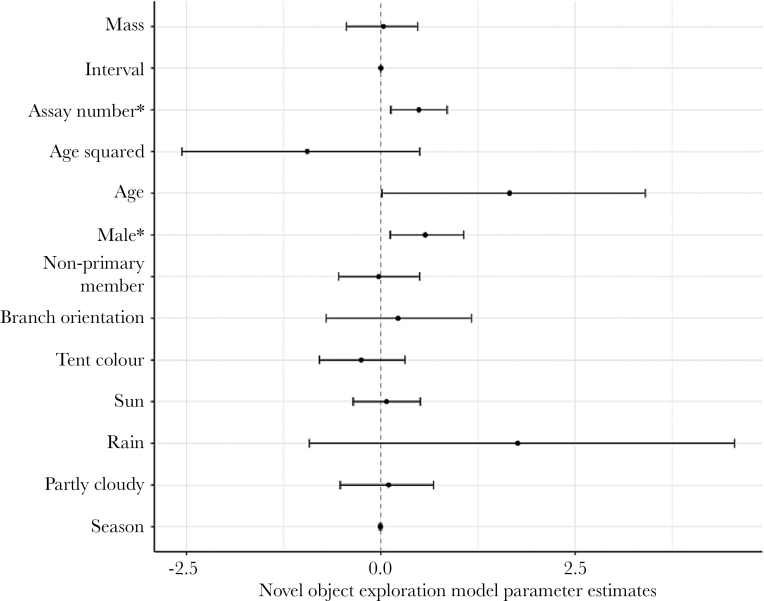
Posterior modes of the fixed effects, and associated 95% credible intervals, in the novel object exploration model: year (contrast 2013), mass (mean centred and divided by 2 standard deviations), interval (days between assay), assay number, age (mean centred and divided by 2 standard deviations; quadratic and linear terms), sex (male = 96, female = 81; contrast = female), social status (primary = 141, non-primary member = 99; contrast = primary), branch orientation (diagonal = 205, vs. parallel = 35, contrast = diagonal), tent colour (blue = 76, vs. green = 164, contrast = blue), weather (cloudy = 167, partly cloudy = 59, rain = 1, sun = 107; contrast = cloudy) and season (number of days from the first of January or June). * indicates posterior modes where the 95% credible intervals do not overlap zero.

For novel environment exploration, exploration scores increased with increasing assay number and age, whereas they were lower when measured in the green rather than the blue tent (Figure 2). There was also a tendency for non-primary members to be slower explorers than primary members (Figure 2). Novel object exploration scores increased with increasing assay number, but unlike novel environment exploration there was a sex-specific effect, such that males explored more than females. There was no effect of mass, interval, age, social status, branch orientation, tent colour, weather or season (Figure 3).

Obstinacy decreased with increasing assay number but was higher when it was partly cloudy than at sunset, and higher in 2013 and 2014 than in 2010 (Supplementary Figure S6). Finally, for escape behaviour, individuals took longer to fly from the perch when it was raining than when it was cloudy, with increasing assay number, in 2013 and 2014 than 2010, with increasing body mass, and when released from the perch rather than the hand (Supplementary Figure S7).

### Heritability

The heritability of behavior, *h*^2^_B_, estimates were moderate for novel environment (0.17, Table 1), and negligible for novel object exploration (<0.01, Table 1). The estimates of the heritability of behaviour excluding observer variance, *h*^2^_B-O_, were moderate for novel environment exploration (0.25, Table 1), and negligible for novel object exploration (<0.01, Table 1). The heritability of personality, *h*^2^_P_, estimates were high for novel environment exploration (0.65, Table 1), and negligible for novel object exploration (0.01, Table 1). The evolvability estimates, *I*_A_, were low for novel environment exploration (<0.01, Table 1) and low for novel object exploration (<0.01, Table 1). Permanent environment effects were negligible for both novel environment exploration and novel object exploration (<0.01 and <0.01, respectively, Table 1). Maternal effects were negligible for novel environment exploration and novel object exploration (<0.01 and <0.01, respectively, Table 1). Observer effects were also low for novel environment exploration and negligible for novel object exploration (0.04 and <0.01, respectively, Table 1).

**Table 1:** The heritability of behaviour (*h*^2^_B_ = *V*_A_ / *V*_P_), heritability of behaviour excluding observer variance (*h*^2^_B-O_*= V*_A_ / *V*_A_ + *V*_PE_ + *V*_M_ + *V*_Res_)), heritability of personality (*h*^2^_P_ = *V*_A_ / (*V*_A_ + *V*_PE_ + *V*_M_)), permanent environment effect (*pe*^2^ = *V*_PE_ / *V*_P_), maternal effect (*m*^*2*^ = *V*_M_ / *V*_P_), observer effect (*obs*^*2*^ = *V*_O_ / *V*_P_), residual effect (*res*^*2*^ = *V*_Residual_ / *V*_P_), additive genetic variance (*V*_A_), total phenotypic variance (*V*_P_) and evolvability (*I*_A_*= V*_A_*/trait mean*^2^), for each moderately repeatable personality trait

Personality trait	*h* ^2^_B_	*h* ^2^_B-O_	*h* ^2^_P_	*pe* ^2^	*m* ^2^	*o* ^2^	*res* ^2^	*V*_A_	*V*_P_	*I*_A_
Novel environment exploration	0.17 (3e-4–0.33)	0.25 (4e-3–0.37)	0.65 (0.09–0.87)	<0.01 (2e-4–0.21)	<0.01 (1e-4–0.07)	0.04 (1e-3–0.23)	0.55 (0.40–0.71)	0.28 (6e-3–0.53)	1.36 (1.11–1.74)	<0.01 (2e-6–1e-2)
Novel object exploration	<0.01 (7e-4–0.37)	<0.01 (7e-4– 0.41)	0.01 (1e-4–0.81)	<0.01 (5e-4–0.45)	<0.01 (2e-4–0.12)	<0.01 (2e-4–0.39)	0.46 (0.19–0.72)	0.01 (0.02–0.81)	2.07 (1.39–4.23)	<0.01 (1.9e-7–5.9e-3)

95% Credible intervals are in brackets.

### Behavioural correlation

A positive among-individual correlation existed between the novel object and novel environment exploration (0.51, 95% credible Interval [Cr.I.] = 0.13–0.68, *n* = 177).

## DISCUSSION

We have shown that novel environment exploration and novel object exploration are moderately repeatable (0.23–0.37), comparable to the mean reported repeatability of behaviour (mean for field and laboratory studies: 0.37, SE = 0.01, mean for field studies: 0.39, SE = 0.01, Bell et al. 2009). We have also shown that personality is heritable in a natural population of a cooperative breeder. More specifically, we have shown that novel environment exploration has a moderate *h*^2^_B_ (0.17), compared to the mean reported *h*^2^_B_ (0.26, SE = 0.01, van Oers and Sinn 2013), and a low *I*_A_ (<0.01) similar to estimates observed for behavioural traits (Hansen et al. 2011). These results reveal that there is a genetic basis for novel environment exploration. However, the low *I*_A_ estimate reveals that novel environment exploration has low evolutionary potential in the population. Novel object exploration, on the other hand, had a negligible *h*^2^_B_ (<0.01), and a low *I*_A_ (<0.01). Low heritability estimates are expected when directional selection has depleted the genetic variation in traits linked to fitness (Falconer and Mackay 1996; Kruuk et al. 2002). Alternatively a low heritability estimate may occur if novel object exploration has a complex genetic architecture (integration of many morphological and behavioural components), then the residual variance and the additive genetic variance may co-vary, and restrict their independent direct effects on heritability (Stirling et al. 2002) or due to lack of power.

Our heritability estimates increased for novel environment exploration (0.17 vs 0.25 vs 0.67) and novel object exploration (<0.01 vs 0.01) when measurement error and temporary environmental effects were, in turn, excluded to estimate *h*^2^_B-O_ and *h*^*2*^_P_. This illustrates how measurement error can confound behaviour measures, and cause the underestimation of *h*^2^_B_ and overestimation of *h*^2^_P_, and that *h*^2^_P_ may be excluding biologically relevant within-individual variation. By excluding measurement error from the heritability estimates of personality, we are better able to estimate the ratio of additive genetic variance to other biologically relevant variance components that contribute to personality.

Observer effects in our study were negligible for novel object and low for novel environment exploration, with observers differing most in their ability to measure novel environment exploration. It is crucial to account for observer effects and other confounding variables in behavioural studies (e.g. Altmann 1974). Permanent environmental effects in our study were also negligible for both traits, although they have been more substantial in other studies (Taylor et al. 2012; Poissant et al. 2013; Petelle et al. 2015). Particularly in territorial species, it is postulated that territory quality can represent such a permanent environmental effect, leading to long-term consequences on personality (Taylor et al. 2012; Petelle et al. 2015). Maternal effects were also negligible for the 2 traits in our study, which is in line with most other studies on the heritability of personality traits in wild populations (Duckworth and Kruuk 2009; Réale et al. 2009; Blumstein et al. 2010; Poissant et al. 2013). However, recent work has found that maternal effects, possibly through early hormonal exposure, can explain some of the variation in personality (Taylor et al. 2012; Petelle et al. 2015), and maternal effects have been shown to have long-term fitness consequences in our study species (Brouwer et al. 2007). Since permanent environment and maternal effects were negligible, we note that we cannot rule out a lack of power in estimating these sources of variation. Furthermore, indirect genetic effects, such as the social partner (Bijma 2014), and sex (Schuett et al. 2010) can also contribute to the heritable variance available for selection, however we did not have the power to test for these effects. Thus, where possible, social genetic, maternal and permanent environmental effects should be accounted for in personality research to avoid a confounded estimate of *V*_A_ (Kruuk and Hadfield 2007; Taylor et al. 2012; Petelle et al. 2015).

Novel object exploration and novel environment exploration had a positive among-individual correlation. This result is similar to previous personality research where fast exploration of a novel environment was associated with a faster approach to a novel object (Verbeek et al. 1994). Furthermore, fast exploratory behaviour has been found to be associated with greater levels of aggression and the formation of routines (Verbeek et al. 1996). Behavioural correlations may constrain behavioural plasticity and cause behavioural carryovers across situations (Sih et al. 2004a), and could explain the maintenance of personality, particularly when the behaviours appear sub-optimal. Trade-offs between correlated behaviours and life-history traits (e.g. growth rate), could then lead to traits being selected together, through correlated selection, so shaping the ecological and evolutionary patterns of personality (Stamps 2007).

Showing that exploratory behaviour is repeatable and heritable in the Seychelles warbler allows for the further investigation of the social environment as a mechanism that generates and maintains personality through state-dependency. The asset protection principle suggests consistent behavioural differences are encouraged through trade-offs with future fitness expectations and survival probability, whereby individuals with a high future reproductive state are predicted to be consistently slow explorers and risk averse, in order to reduce their risk of mortality from predation (Wolf et al. 2007). The social niche hypothesis further suggests that, when individuals repeatedly interact with one another, individuals benefit through reduced social conflict by developing social niches. These social niches, such as breeding roles, cause individuals to behave differently (although the direction of the relationship is still unclear) and encourages behavioural consistency due to social conflict and the costs incurred by changing social niches (Bergmüller and Taborsky 2010). Although we have shown that social status (primary or non-primary) is not a mechanism that generates and maintains these individual differences in the Seychelles warbler, we have shown that exploration of a novel object is reproductive state-dependent, whereby young individuals with low future reproductive potential exhibited fast exploratory behaviour (Edwards et al. 2016). Exploratory behavioural variation may therefore be generated and maintained by reproductive tactics that are modified to suit environmental conditions (Hammers et al. 2013).

## SUMMARY

In summary, we have shown that there is a genetic basis to personality in a natural population of a cooperatively breeding species. This provides further understanding of the potential variance available for selection in this system and suggests that state may be a mechanism that generates and maintains personality. Further studies should investigate the selective processes that create these individual differences in behaviour and the implications they have in a cooperatively breeding environment.

## SUPPLEMENTARY MATERIAL

Supplementary data are available at *Behavioral Ecology* online.

## FUNDING

This work was supported by a Natural Environment Research Council studentship (X/007/001-15 to HAE), a Natural Environment Research Council fellowship (NE/I021748/1 to HLD), 2 Schure Beijerinck Popping grants (SBP2013/46 to HAE and SBP2012/26 to HLD) and TB was supported by a Leverhulme Fellowship.

## Supplementary Material

Heritability_Supp_BehavEco
