# Supplementary material for: Repeatable and heritable behavioural variation in a wild cooperative breeder
Source: Behav Ecol. 2017 Feb 18;28(3):668–76. doi: 10.1093/beheco/arx013 (PMC5873258; doi:10.1093/beheco/arx013)

**Supporting material**

**Repeatable and heritable behavioural variation in a wild cooperative breeder**

**Supplementary Table S1:** Sample sizes for each personality assay

**Supplementary Table S2:** Sensitivity analysis: The impact of prior specification on estimates of heritability and repeatability

**Supplementary Figure S1:** Box-plot of latency time to move in the novel object exploration and control assay

**Supplementary Figure S2:** Box-plot of stick touches in the novel object exploration and control assay

**Supplementary Figure S3:** Box-plot of exploration scores in the novel object exploration and control assay

**Supplementary Figure S4**: A power analysis, using the R package pedantics 1.5, to detect heritability of exploration of a novel environment.

**Supplementary Figure S5**: A power analysis, using the R package pedantics 1.5, to detect heritability of novel object exploration.

**Supplementary Figure S6:** Forest plot of the repeatability model for obstinacy

**Supplementary Figure S7:** Forest plot of the repeatability model for escape response

# Supplementary Table S1: The number of birds with single or repeat samples for each personality assay.

|  | **Total birds assayed** | **Number of birds with the following samples** | | | | | | |
| --- | --- | --- | --- | --- | --- | --- | --- | --- |
| **Assay** |  | **1** | **2** | **3** | **4** | **5** | **6** | **7** |
| Obstinacy | 300 | 153 | 100 | 31 | 7 | 7 | 1 | 1 |
| Novel environment exploration | 312 | 175 | 96 | 25 | 8 | 5 | 3 | 0 |
| Novel object exploration | 177 | 120 | 52 | 4 | 1 | 0 | 0 | 0 |
| Escape response | 300 | 165 | 90 | 29 | 7 | 8 | 0 | 1 |

**Supplementary Table S2:** Sensitivity analysis: The impact of prior specification on estimates of repeatability and heritability. Prior specifications: Inverse gamma: R = (V = 1, nu = 0.002), G = (V = 1, nu = 0.002); Modified inverse gamma prior: R = (V = 1, nu = 0.02), G = (V = 1, nu = 0.02); Expanded prior: R = (V = 1, nu = 0.002), G=V = 1, n = 0.002, alpha.mu = 0 and alpha.V = 1000; Modified expanded prior: R = (V = 1, nu = 0.02), G=V = 1, n = 0.02, alpha.mu = 0 and alpha.V = 1000.

| **Trait** | **Estimate** | **Prior** | **Fixed effects** | **Estimate** | **V_A_** | **V_P_** |
| --- | --- | --- | --- | --- | --- | --- |
| Obstinacy | Repeatability | Expanded | Year+mass+interval+assay number+age+age^2^+sex+social status+weather+season | <0.01 (0–8.5e-4) | NA | 4.35 (3.47–5.82) |
| Obstinacy | Repeatability | Modified expanded | Year+mass+interval+assay number+age+age^2^+sex+social status+weather+season | <0.01 (0– 9.1e-4) | NA | 4.52 (3.48–6.0) |
| Novel environment exploration | Repeatability | Inverse gamma | Mass+interval+assay number+age+age^2^+sex+social status+branch orientation+release method+tent colour+weather+season | 0.23 (0.08– 0.36) | NA | 1.33 (1.10– 1.84) |
| Novel environment exploration | Repeatability | Modified inverse gamma | Mass+interval+assay number+age+age^2^+sex+social status+branch orientation+release method+tent colour+weather+season | 0.22 (0.09– 0.36) | NA | 1.35 (1.12– 1.81) |
| Novel environment exploration | Heritability | Inverse gamma | Mass+interval+assay number+age+age^2^+sex+social status+branch orientation+release method+tent colour+weather+season | 0.17  (3.4e-4–0.33) | 0.28 (5.7e-3–0.53) | 1.36 (1.11–1.74) |
| Novel environment exploration | Heritability | Modified inverse gamma | Mass+interval+assay number+age+age^2^+sex+social status+branch orientation+release method+tent colour+weather+season | 0.13  (5.8e-3–0.32) | 0.20 (5.7e-3–0.51) | 1.32 (1.14–1.75) |
| Novel object exploration | Repeatability | Expanded | Mass+interval+assay number+age+age^2^+sex+social status+branch orientation+tent colour+weather+season | 0.37 (0.04– 0.59) | NA | 2.01 (1.39– 4.29) |
| Novel object exploration | Repeatability | Modified expanded | Mass+interval+assay number+age+age^2^+sex+social status+branch orientation+tent colour+weather+season | 0.35 (0.03– 0.57) | NA | 1.96 (1.45– 4.16) |
| Novel object exploration | Heritability | Expanded | Mass+interval+assay number+age+age^2^+sex+social status+branch orientation+tent colour+weather+season | <0.01 (7e-4–0.37) | 0.01 (0.02–0.81) | 2.07 (1.39–4.23) |
| Novel object exploration | Heritability | Modified expanded | Mass+interval+assay number+age+age^2^+sex+social status+branch orientation+tent colour+weather+season | <0.01 (4.45e-8–0.39) | 0.01 (1.1e-7–1.04) | 2.19 (1.55–4.07) |
| Escape response | Repeatability | Expanded | Year+mass+interval+assay number+age+age^2^+sex+social status+method+weather+season | <0.01 (1.02e-9–0.14) | NA | 10.59 (7.37–18.05) |
| Escape response | Repeatability | Modified expanded | Year+mass+interval+assay number+age+age^2^+sex+social status+method+weather+season | <0.01 (3.7-12– 0.15) | NA | 10.18 (7.51– 19.69) |
| Novel environment/Novel object bivariate | Among individual correlation | V = diag (2), n=1.002 | Mass+interval+assay number+age+age^2^+sex+social status+branch orientation+tent colour+weather+season | 0.51 (0.13–0.68) | NA | NA |
| Novel environment/Novel object bivariate | Among individual correlation | V = diag (2), n=1.02 | Mass+interval+assay number+age+age^2^+sex+social status+branch orientation+tent colour+weather+season | 0.54 (0.14–0.77) | NA | NA |

**Figures**

**Supplementary Figure S1**: The latency time (sec) to move once the novel object (N = 185) or tree branch with no novel object (control; N = 185) is inserted into the tent. The black dot is the median latency time; the top and bottom of the box represent the first and third quartiles, respectively; the top and bottom whiskers represent the highest and lowest values excluding outliers, respectively; and, the blue circles are outliers.

**
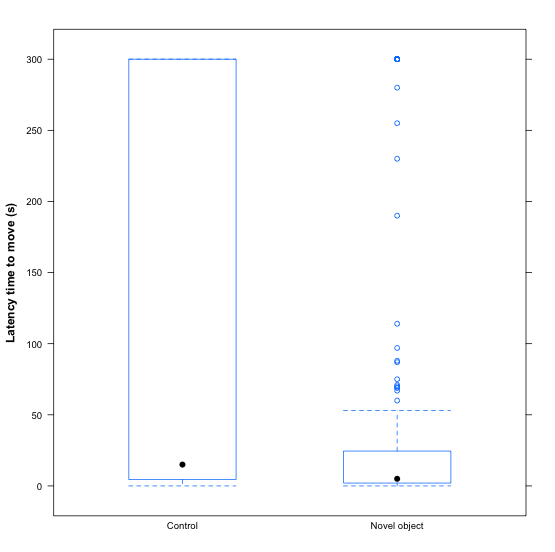
**

**Supplementary Figure S2**: The number of touches to the tree branch in the control (no novel object attached; N = 185) and novel object assay (tree branch with novel object attached; N = 185). The black dot is the median number of touches; the top and bottom of the box represent the first and third quartiles, respectively; the top and bottom whiskers represent the highest and lowest values excluding outliers, respectively; and, the blue circles are outliers.

**
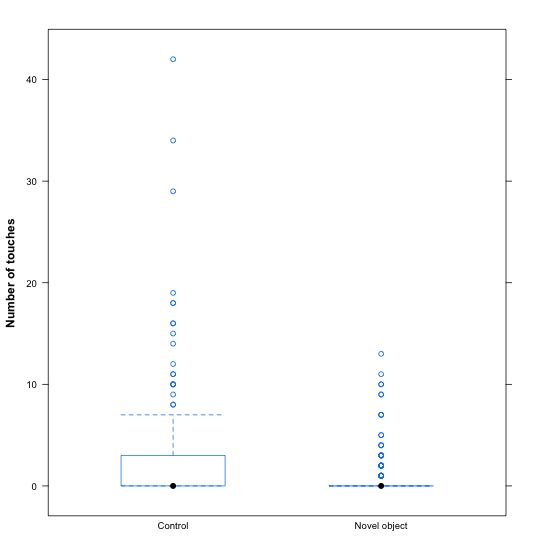
**

**Supplementary Figure S3**: Exploration of the novel object score (sum of the number of hops, flights and trees visited) in the control (N = 185) and novel object assay (N = 185). The black dot is the median behaviour score; the top and bottom of the box represent the first and third quartiles, respectively; the top and bottom whiskers represent the highest and lowest values excluding outliers, respectively; and, the blue circles are outliers.

**
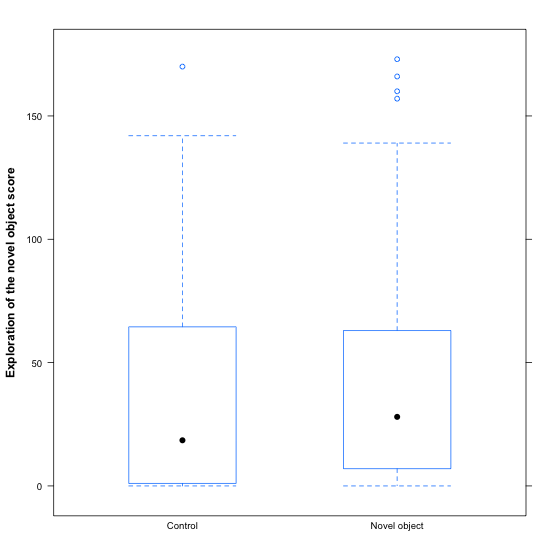
**

**Supplementary Figure S4**: A power analysis using pedantics 1.5. The scatterplot shows the power of detecting the true heritability of exploration of a novel environment, where true heritability was simulated from 0.00 to 0.5, in incremental steps of 0.01. A pruned pedigree (containing only individuals informative for exploration of a novel environment) was used. The dotted horizontal line shows power ≥ 0.8.

**
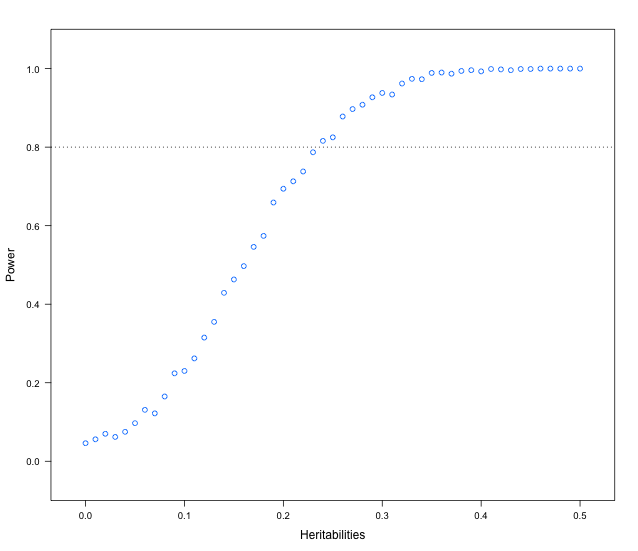
**

**Supplementary Figure S5**: A power analysis using pedantics 1.5. The scatterplot shows the power of detecting the true heritability of novel object exploration, where true heritability was simulated from 0.00 to 0.5, in incremental steps of 0.01. A pruned pedigree (containing only individuals informative for novel object exploration) was used. The dotted horizontal line shows power ≥ 0.8.

**
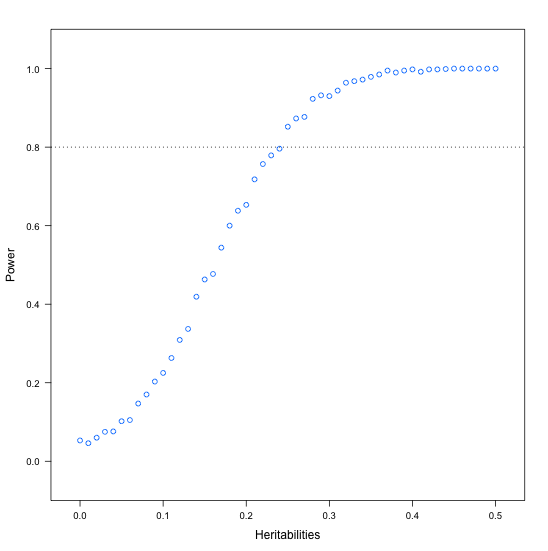
**

**Supplementary Figure S6**: Estimates of the posterior mode distributions of the fixed effects in the obstinacy model: year* (2012 = 200, 2013 = 200, 2014 = 57; contrast level = 2010), mass (mean centred and divided by two standard deviations), interval (days between assay), assay number*, age (mean centred and divided by two standard deviations; quadratic and linear terms), sex (male = 149, female = 151; contrast = female), social status (primary = 294, non-primary member = 228; contrast = primary), weather* (partly cloudy = 118, cloudy = 124, rain = 7, sun = 265; contrast = sunset) and season (number of days from the first of January or June). * indicates posterior modes where the 95% credible intervals do not overlap zero.


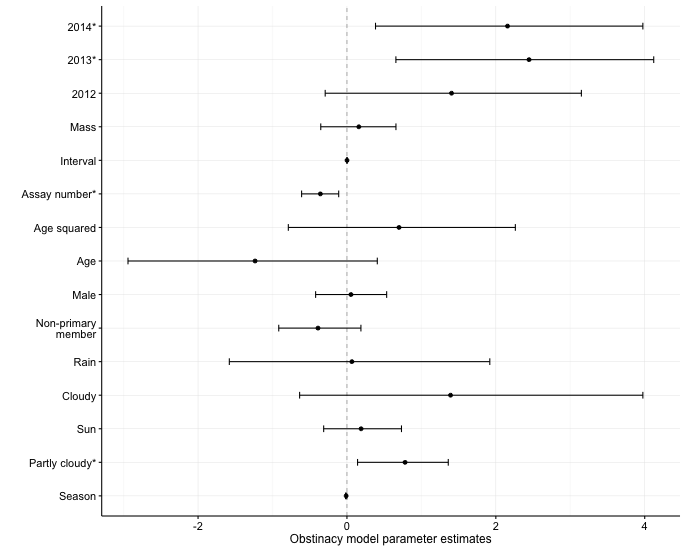


**Supplementary Figure S7**: Estimates of the posterior mode distributions of the fixed effects in the escape response model: year* (2012 = 195, 2013 = 195, 2014 = 54; contrast = 2010), mass* (mean centred and divided by two standard deviations), interval (days between assay), assay number*, age (mean centred and divided by two standard deviations; quadratic and linear terms), sex (male = 149, female = 151; contrast = female), social status (primary = 284, non-primary member = 223; contrast = primary), method (hand = 259, perch = 248; contrast = hand), weather * (partly cloudy = 111, cloudy = 111, sun = 244, sunset = 8; contrast = rain) and season (number of days from the first of January or June). * indicates posterior modes where the 95% credible intervals do not overlap zero.


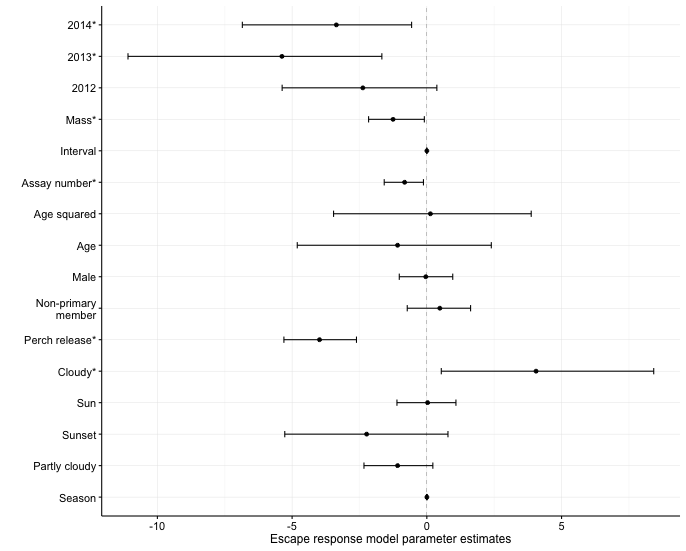

Supplement: Heritability_Supp_BehavEco [file arx013_suppl_heritability_supp_behaveco.docx]
